# The Conservation and Application of Three Hypothetical Protein Coding Gene for Direct Detection of *Mycobacterium tuberculosis* in Sputum Specimens

**DOI:** 10.1371/journal.pone.0073955

**Published:** 2013-09-13

**Authors:** Lianhua Qin, Shihui Gao, Jie Wang, Ruijuan Zheng, Junmei Lu, Zhongyi Hu

**Affiliations:** Shanghai Key Laboratory of Tuberculosis, Shanghai Pulmonary Hospital, Tongji University School of Medicine, Shanghai, China; St. Petersburg Pasteur Institute, Russian Federation

## Abstract

**Background:**

Accurate and early diagnosis of tuberculosis (TB) is of major importance in the control of TB. One of the most important technical advances in diagnosis of tuberculosis is the development of nucleic acid amplification (NAA) tests. However, the choice of the target sequence remains controversial in NAA tests. Recently, interesting alternatives have been found in hypothetical protein coding sequences from mycobacterial genome.

**Methodology/Principal Findings:**

To obtain rational biomarker for TB diagnosis, the conservation of three hypothetical genes was firstly evaluated in 714 mycobacterial strains. The results showed that SCAR1 (Sequenced Characterized Amplified Region) based on Rv0264c coding gene showed the highest conservation (99.8%) and SCAR2 based on Rv1508c gene showed the secondary high conservation (99.7%) in *M. tuberculosis* (MTB) strains. SCAR3 based on Rv2135c gene (3.2%) and IS6110 (8%) showed relatively high deletion rate in MTB strains. Secondly, three SCAR markers were evaluated in 307 clinical sputum from patients in whom TB was suspected or patients with diseases other than TB. The amplification of IS6110 and 16SrRNA sequences together with both clinical and bacteriological identification was as a protocol to evaluate the efficacy of SCAR markers. The sensitivities and specificities, positive predictive value (PPV) and negative predictive value (NPV) of all NAA tests were higher than those of bacteriological detection. In four NAA tests, IS6110 and SCAR3 showed the highest PPV (100%) and low NPV (70% and 68.8%, respectively), and SCAR1 and SCAR2 showed the relatively high PPV and NPV (97% and 82.6%, 95.6% and 88.8%, respectively).

**Conclusions/Significance:**

Our result indicated that SCAR1 and SCAR2 with a high degree of sequence conservation represent efficient and promising alternatives as NAA test targets in identification of MTB. Moreover, the targets developed from this study may provide more alternative targets for the development of a multisite system to effectively detect MTB in samples.

## Introduction

Tuberculosis remains a significant threat to human health for thousands of years. Even today, infection with the etiologic agent, *Mycobacterium tuberculosis* (MTB), is associated with extraordinarily high rates of morbidity and mortality worldwide. Accurate and early detection of pathogenic mycobacteria in clinical samples is of major importance in the control of human tuberculosis. Delay in diagnosis will result in late initiation of therapy and prolonged transmission. Conventional diagnosis of tuberculosis mainly relies on the acid-fast staining of bacilli smears and mycobacterial culture of sputa from TB patients. However, bacteriological diagnosis is time-consuming, because culturing of *M. tuberculosis* can take 4 to 8 weeks. Direct staining and microscopy of clinical specimens lack sensitivity and specificity [1].

More recently, nucleic acid amplification (NAA) tests by different PCR technology for the successful identification of MTB directly from clinical samples, such as sputum, blood, urine, and cerebrospinal or pleural fluid have been published [2]–[4]. These nucleic acid-based methods have been found to be more sensitive than conventional methods and were able to detect even small numbers of MTB cells [1], [5]. At present, a number of studies related NAA tests have been designed to detect MTB [6]–[10]. However, in-house tests vary widely in their accuracy. Results from different clinical studies evaluating the various amplification assays were divergent concerning their specificity and sensitivity [7], [8]. Factors affecting NAA test accuracy were identified using meta-analytical methods, and the results showed that amplified targets and protocols used to amplify mycobacteria DNA in samples are significantly associated with accuracy [11], [12]. While protocols have been greatly optimized, the choice of the target sequence remains controversial. The lack of targets in some strains may be the one of main reasons causing much lower and highly variable detecting accuracy in clinical diagnosis [13], [14]. A possible solution may be to identify more effective target or use more than one target to increase detection accuracy.

The genome of MTB (H37Rv) contains 4,019 protein coding genes, of which more than thousand have been categorized as unknown function or are classified as conserved hypothetical proteins [15]. Though these hypothetical protein coding genes have been little studied in the TB diagnosis, some have been used as DNA target sequences in the detection of MTB [8], [16]. In this study, we firstly evaluated the conservation of three hypothetical genes, which code conserved hypothetical protein Rv0264c, Rv1508c, and Rv2135c, in clinical strains of MTB, and then tested the application for direct detection of MTB in sputum specimens.

## Materials and Methods

### Ethics Statement

All these patients were treated in accordance with the Helsinki Declaration on the participation of human subjects in medical research. The ethics approvals were obtained for this study from Shanghai Pulmonary Hospital Ethics Committee (the permit numbers: 2011-fk-03). A written informed consent was obtained from each of participants.

### Mycobacterial Strains and Preparation

A total of 714 mycobacterial strains were used to evaluate the conservation of three coding genes, including 590 MTB strains (collected between May 2011 and November 2012 from regions of Eastern China, including Shanghai, Jiangsu, Zhejiang, Shandong, Fujian, Anhui, and Jiangxi, by Laboratory of Tuberculosis, Shanghai Pulmonary Hospital), 3 other *Mycobacterium tuberculosis* complex (MTC) strains *M. africanum* (ATCC25420), *M. bovis* (ATCC27294), BCG (Danish), 19 nontuberculous mycobacterial (NTM) species *M. abscessus* (ATCC19977), *M. chelonae* (ATCC14472), *M. nonchromogenicum* (ATCC19530), *M. scrofulaceum* (ATCC19981), *M. intracellulare* (ATCC13950), *M. avium* (ATCC25291), *M. gordonae* (ATCC14470), *M. flavescens* (ATCC 14474), *M. marinum* (ATCC927), *M. terrae* (ATCC19619), *M. fortuitum* (ATCC6481), *M. phlei* (ATCC11758), *M. kansasii* (ATCC12478), *M. aurum* (ATCC23366), *M. aichiense* (ATCC27280), *M. parafortuitum* (ATCC19686), *M. malmoense* (ATCC29571), *M. smegmatis* (ATCC19420), *M. neoaurum* (ATCC25795), and 102 NTM isolates (isolated from Guangzhou Chest Hospital). All strains were grown in Sauton culture medium (0.5 g/L KH_2_PO_4_, 0.5 g/L MgSO4.7H_2_O, 2 g/L citric acid, 0.05 g/L ferric ammonium citrate, 4.0 g/L L-asparagine, 6% glycerol and 0.02% Tween 80). Cells were sterilized at 80°C for 30 min, and were harvested by centrifugation (12,000×g for 5 min). The bacterial pellet was washed three times with sterilized saline and re-collected by centrifugation (12,000×g for 10 min each time). Aliquots of the bacterial pellet were frozen at −20°C until they were processed for PCR.

### Sputum Samples and Preparation

307 cases of clinical sputum specimens were obtained from patients with respiratory symptoms at Shanghai Pulmonary Hospital (Shanghai, PR China) to test the clinical applicability of three coding genes as target DNA in TB diagnosis. This included 203 samples from patients in whom TB was suspected and 104 specimens from patients with diseases other than TB. The diagnosis of TB was made on the basis of (i) TB symptoms and signs; (ii) TB lesion detected by chest X-ray or computed tomography scan; (iii) tuberculin skin test (0.1 to 100 tuberculin units); (iv) symptom relief from anti-TB treatment. Sputum samples were decontaminated by the standard protocol with N-acetyl-L-cysteine–2% NaOH and were concentrated by centrifugation at 3,000×g for 20–30 min [17]. The sediment was resuspended in 2 mL of supernatant.

### Conventional Identification

All sputum specimens were processed for direct smear examination by fluorescence microscopy after auramine-rhodamine staining and were cultured onto three Loewenstein-Jensen slants. Mycobacterial cultures were incubated at 37°C for 8 weeks and were examined weekly for growth. Positive slides were confirmed by Ziehl-Neelsen staining. Bacterial colonies were simultaneously identified as *M. tuberculosis* or other different species of mycobacteria by 16SrRNA sequencing [18] and conventional methods, including growth characteristics, pigment production, colony morphology, and growth in the presence of PNB (p-nitrobenzoic acid) and TCH (thiophene-2-carboxylic acid hydrazide). The exact methods used in our laboratory were in accord with those described in Chinese Laboratory Science Procedure of Diagnostic Bacteriology in Tuberculosis. Aliquots of the resuspended sediments of sputum were frozen at −20°C until they were processed for PCR.

### Extraction of DNA

The total of 50 µL resuspended bacterial pellet or 100 µL resuspended sputum sediments were treated with DNA lysis buffer (10 mmol/L NaCl, 1 mg/mL SDS, 15% Chelex-100, 1% Tween 20). The mixture was incubated at 50°C for 1 h, followed by 100°C for 10 min, then centrifuged (5,000×g for 10 min) to obtain the aqueous phase containing genomic DNA that was used for PCR amplification.

### PCR Assays in Clinical Strains

All genomic DNA were respectively amplified by 16SrRNA gene [9], IS6110 element [10] and three SCAR (Sequenced Characterized Amplified Region) markers based on Rv0264c (named SCAR1), Rv1508c (named SCAR2), and Rv2135c (named SCAR3) coding gene. The primers of NAA targets were listed in Table 1.

**Table 1:** The primers of NAA test in the identification of *Mycobacterium* strains.

Gene name	Primer sequence	Product length	Annealing temperature	Organism identification
16SrRNA	5′-GCGTGCTTAACACATGCAA-3′	570 bp	53°C	*Mycobacterium*
	5′-CGCTCACAGTTAAGCCGT-3′			
IS6110	5′-TCAGGTCGAGTACGCCTTCT-3′	874 bp	60°C	MTC
	5′-GGTCGCAGAGATCCGCGGTC-3′			
Rv0264c	5′-CAGCCGCTGACGGGTGA-3′	162 bp	64°C	MTC
	5′-GCGCTGATGCTGCAATGT-3′			
Rv1508c	5′-TGACTAGGACAGCAAACCCGATCT-3′	174 bp	60°C	MTC*
	5′-ACAGTCGATCCACGGCATCATCAAGT-3′			
Rv2135c	5′-ATCTGGACAGCTTTCAGCGAAT-3′	209 bp	60°C	MTC
	5′-AGTCGTGCACCGCCTGTA-3′			

* MTC except *M. bovis* and BCG.

The PCR reaction of mixtures (20 µL) consisted of 1×taq PCR buffer, 2.5 mmol/L MgCl_2_, 0.2 mmol/L dNTP, 0.2 umol/L primer 1, 0.2 umol/L primer 2, 1 U Taq DNA polymerase, 10 ng of template DNA. The reaction conditions were the following: 94°C for 5 min, followed by 35 cycles of 94°C for 1 min, annealing temperature (Table 2) for 30 s, 72°C for 1 min, and finally 72°C for 10 min. All PCR products of 714 mycobacterial strains were electrophoresed on 2% agarose gel with ethidium bromide staining. PCR products of SCAR markers from some strains were randomly selected and sequenced to exclude fake positive. The clinical strains with inconsistent diagnosis among NAA test and traditional bacteriological examinations tests were needed to sequence their PCR products of 16SrRNA gene. The 570 bp of 16SrRNA sequence was compared to those deposited in the GenBank database using basic Local alignment search tool on the Internet (http://www.ncbi.nlm.nih.gov/BLAST/). A 99% identity was used to define a specific species.

**Table 2:** Efficiency of three molecular markers in the identification of *M. tuberculosis* evaluated by 714 various *Mycobacterium* strains.

Group	No. of strains	16sRNA	IS6110	SCAR1^a^	SCAR2^b^	SCAR3^c^	Organism identification
I	532	+	+	+	+	+	*M. tuberculosis*
	8	+	+	+	+	−	*M. tuberculosis*
	2	+	+	−	+	+	*M. tuberculosis*
	1	+	+	+	−	+	*M. tuberculosis*
	36	+	−	+	+	+	*M. tuberculosis*
	11	+	−	+	+	−	*M. tuberculosis*
II	1	+	+	−	+	+	*M. bovis*
	1	+	+	−	+	+	BCG (Danish)
III	1	+	+	+	+	+	*M. africanum*
IV	23	+	−	−	−	−	*M. abscessus*
	18	+	−	−	−	−	*M. chelonae*
	14	+	−	−	−	−	*M. nonchromogenicum*
	12	+	−	−	−	−	*M. scrofulaceum*
	10	+	−	−	−	−	*M. intracellulare*
	10	+	−	−	−	−	*M. avium*
	9	+	−	−	−	−	*M. gordonae*
	6	+	−	−	−	−	*M. flavescens*
	5	+	−	−	−	−	*M. marinum*
	2	+	−	−	−	−	*M. terrae*
	3	++	−	−	−	−	*M. fortuitum*
	2	+	−	−	−	−	*M. phlei*
	1	+	−	−	−	−	*M. kansassi*
	1	+	−	−	−	−	*M. aurum*
	1	+	−	−	−	−	*M. aichiense*
	1	+	−	−	−	−	*M. parofortuitum*
	1	+	−	−	−	−	*M. malmoense*
	1	+	−	−	−	−	*M. smegmatis*
	1	+	−	−	−	−	*M. neoaurum*

a SCAR1 based on Rv0264c coding gene.

b SCAR2 based on Rv1508c coding gene.

c SCAR3 based on Rv2135c coding gene.

### Real-time PCR in Sputum Specimens

The application of three coding genes for direct detection of MTB in sputum specimens was analyzed by a 7500 real-time PCR system (Applied Biosystems). For 1 µL of DNA, we added 12.5 µL of PCR master mix (Quantitect SYBR green PCR buffer (Tris-Cl, KCl, (NH_4_)_2_SO_4_, 5 mmol/L MgCl_2_ (pH 8.7)), dNTP, SYBR green I, and ROX), 0.5 µL forward primer (10 pmol/µL), 0.5 µL reverse primer (10 pmol/µL), and 10.5 µL ddH_2_O. Standard curves of quantified and diluted PCR products, as well as negative controls, were included in each PCR run.

## Results

### Amplification of DNA from Mycobacterium Strains by PCR

All chromosomal DNA extracted from 714 mycobacterium strains including 21 reference strains and 693 clinical strains diagnosed by traditional bacteriological tests were amplified and tested by Mycobacterium genus-specific primers (F1, R1) from the 16SrRNA gene fragment, MTC-specific primers (IS52, IS57) derived from the IS6110 insertion sequence, and the SCAR markers developed from three coding genes Rv0264c, Rv1508c and Rv2135c. The results were summarized in Table 2. All 714 strains from mycobacterium accurately produced the 570 bp 16SrRNA amplicon. All 121 NTM strains (19 NTM reference strains and 102 NTM clinical isolated strains) were negative by both IS6110-PCR and three SCAR markers amplification. In 590 MTB clinical strains, the positive rate of IS6110, SCAR1, SCAR2 and SCAR3 markers was 92% (543/590), 99.8% (559/590), 99.7% (588/590), and 96.8% (571/590), respectively. The deletion rate of IS6110 sequence was the highest in 4 NAA targets. SCAR1 and SCAR2 showed relatively high conservation in MTB strains. Though SCAR3 showed relatively high deletion rate, its conservation in MTB strains was higher than IS6110 sequence. Other three MTC strains were all positive by IS6110, SCAR1 and SCAR3 amplification. SCAR2-PCR was positive in *M. africanum* but negative in *M. bovis* and BCG, which showed that Rv1508c may be absent in *M. bovis* species.

### Culture and Direct Microscopy

All 307 samples were detected by Ziehl-Neelsen staining and growth in Loewenstein-Jensen medium. Acid-fast bacilli was detected in 138 samples after Ziehl-Neelsen. Growth of mycobacteria in Loewenstein-Jensen medium was detected in 151 samples, in which 129 were identified as *M. tuberculosis*, 15 were identified as nontuberculous mycobacterial species (2 *M. kansassi*, 2 *M. chelonae–abscessus*, 10 *M. intracellulare*, 1 *M. avium*), and 7 were identified as pathogenic bacteria other than mycobacterium (Table 3).

**Table 3:** Comparison of PCR with conventional procedures for detection of *M. tuberculosis* in clinical samples.

Group	Smear/culture		PCR-results										Organism identification	Samples origin
			16SrRNA		IS6110		SCAR1		SCAR2		SCAR3			
			P	N	P	N	P	N	P	N	P	N		
I	P/P	101	101	0	96	5	101	0	101	0	91	10	*M. tuberculosis*	TB
	P/N	23	23	0	13	10	19	4	21	2	14	9	*M. tuberculosis*	TB
	N/P	21	21	0	12	9	16	5	18	3	12	9	*M. tuberculosis*	TB
II	N/N	43	27	16	16	27	28	15	33	10	17	26	ND^c^	TB
III	P/P	2	2	0	0	2	0	2	1	1	0	2	*M. kansassi*	TB
		1	1	0	0	1	0	1	0	1	0	1	*M. chelonae -abscessus*	TB
		6	6	0	0	6	0	6	0	6	0	6	*M. intracellulare*	TB
		1	1	0	0	1	0	1	0	1	0	1	*M. avium*	TB
	N/P	4	4	0	0	4	2	2	2	2	0	4	*M. intracellulare*	TB
		1	1	0	0	1	0	1	1	0	0	1	*M. chelonae -abscessus*	TB
IV	N/P	7^a^	0	7	0	7	0	7	0	7	0	7	ND^c^	non-TB
V	N/N	4^b^	0	4	0	4	3	1	4	0	0	4	ND^c^	non-TB
VI	N/N	93	0	93	0	93	0	93	0	93	0	93	ND^c^	non-TB

a Possible false-negative result by culture.

b Possible false-negative result by the SCAR1.

c ND, not determined.

### Comparison of Real-time PCR in Sputum Specimens with Bacteriological and Clinical Data

A total of 307 samples were examined by routine microbiology laboratory techniques and PCR by using the IS6110 sequences and Rv0264c, Rv1508c, Rv2135c genomic fragment as NAAT targets. The results are summarized in Table 4.

**Table 4:** PCR results compared with culture and stain results and clinical assessment of patients.

Test and result	Final interpretation (No. of specimens)		Sensitivity (%)	Specificity (%)	PPV^a^ (%)	NPV^b^ (%)
	Positive	Negative				
Stain						
Positive	124	14	66	88.2	89.9	62.1
Negative	64	105				
Culture						
Positive	129	21	68.6	82.4	86	62.4
Negative	59	98				
IS6110-PCR						
Positive	137	0	72.9	100	100	70
Negative	51	119				
SCAR1-PCR						
Positive	164	5	87.2	95.8	97	82.6
Negative	24	114				
SCAR2-PCR						
Positive	173	8	92	93.3	95.6	88.8
Negative	14	111				
SCAR3-PCR						
Positive	134	0	71.3	100	100	68.8
Negative	54	119				

a PPV, positive predictive value.

b NPV, negative predictive value.

In group I, II, and III, a definitive diagnosis of TB was made on the basis of clinical data. In group I, 101 samples were both smear-positive and culture-positive, and were also positive in SCAR1 and SCAR2 amplification; the positive rate of IS6110 and SCAR3 markers was 95% (96/101), and 90.1% (91/101) in these 101 samples, respectively. 44 samples from group I were smear-positive but culture-negative (n = 23) or smear- negative but culture-positive (n = 21); The positive rate of these samples was 56.8%, 80%, 88.6%, 59.1% in IS6110, SCAR1, SCAR2 and SCAR3 PCR amplification, respectively. The samples from group II (n = 43) were negative in both smear and culture, the positive rate of these samples was relatively low in PCR amplification (37.2% in IS6110-PCR, 65.1% in SCAR1-PCR, 76.7% in SCAR2-PCR, 40% in SCAR3-PCR).

Group III (Table 3) included 15 specimens from patients showing clinical manifestations of a mycobacterial infection. All of these specimens were culture positive, and 66.7% (10/15) of these samples were smear-positive. However, all of these samples were both negative in the IS6110 and SCAR3 amplification, and two sample was positive in SCAR1-PCR and one samples positive in SCAR1-PCR. These samples were diagnosed as having infection with mycobacteria other than *M. tuberculosis* by conventional methods and 16SrRNA sequencing.

Finally, of the 105 samples from patients without mycobacterial infection, 93 were found to be negative in smear, culture, and PCR amplification, as expected (group IV, Table 3); however, of the 11 remaining specimens, 7 were culture-positive but smear and PCR negative (group V, Table 3), which were identified as pathogenic bacteria other than mycobacterium based on 16SrRNA sequencing, and 4 were SCAR1-PCR or SCAR2-PCR positive but 16SrRNA-PCR/IS6110-PCR and culture/smear negative (group VI, Table 3).

### Sensitivity and Specificity

After resolution of the discrepant analysis by comparison of both clinical and bacteriological criteria and patient history, a total of 188 specimens were considered true positive for TB infection (group I, II) and 119 samples were considered true negative for TB disease (including 104 specimens in group IV, V and VI, and the 15 specimens in group III that contained *M. kansassi, M. chelonae–abscessus, M. intracellulare, M. avium*). The sensitivities and specificities of the PCR assay, staining, and culture were evaluated in comparison with the clinical assessment of patients and are summarized in Table 4. In four NAA tests, the positive predictive value (PPV) and negative predictive value (NPV) of IS6110, SCAR1, SCAR2 and SCAR3 markers were 100% and 70%, 97% and 82.6%, 95.6% and 88.8%, 100% and 68.8%, respectively. Though IS6110 and SCAR3 showed the highest PPV, they showed relatively low NPV in four PCR assays.

## Discussion

Acid-fast staining microscopy of specimens combined with isolation and culture of the bacilli remains the “gold standard” method to specifically identify mycobacteria. However, the diagnosis of TB based on bacteriological method is difficult because of the requirement for the identification of *M. tuberculosis* in patient specimens. One of the most important technical advances in diagnosis of tuberculosis is the development of nucleic acid amplification (NAA) tests, which have emerged as promising alternatives to ensure early detection of *M. tuberculosis* in clinical samples.

Reports of studies based on NAA tests for the successful identification of *M. tuberculosis* by different PCR technology have been published [6]–[10], [13], [14]. 16SrRNA gene is the target sequence amplified by commercial kits and the performance of these tests is excellent. However, since the target gene is present in all mycobacterial species, the discrimination between mycobacteria must rely on hybridization with specific DNA probes [18], [19]. The most popular target sequence is the insertion sequence IS6110. Even though IS6110 element with a high copy number in genome provides a good sensitivity for detecting MTB in the meta-analysis by Flores [11], [12], the specificity of IS6110-based assays is controversial. One potential problem with the target is that some strains lack these sequences in its genome, which seriously compromises the use of IS6110-based PCR for tuberculosis diagnosis [14]. Other target sequences have also been developed to identify TB infection by PCR; these include amplification of sequences encoding mycobacterial antigens, and intergenic regions [20]. However, while protocols used to amplify DNA from mycobacteria present in a wide range of samples have been greatly optimized, the choice of the target sequence remains controversial.

Recently, interesting alternatives have found in hypothetical protein coding sequences. The analysis of genome sequencing revealed that there are more than 4000 protein coding genes in the genome of *M. tuberculosis* (H37Rv), of which a considerable fraction of open reading frames is still labeled as conserved hypothetical protein. Except coding sequences as a target for PCR diagnosis, some unknown function proteins have also been revealed as B cell and/or T cell antigens for TB diagnosis [21], [22]. These results implied that rational biomarker for TB diagnosis may be obtained from these conserved hypothetical protein or their coding sequences.

In this study, we first evaluated the conservation of three hypothetical genes, which code conserved hypothetical protein Rv0264c, Rv1508c, and Rv2135c, in clinical strains of *M. tuberculosis*. The results showed that three hypothetical protein coding sequences had higher conservation than insertion sequence IS6110 in tested MTB strains. SCAR1 based on Rv0264c coding gene showed the highest positive rate (99.8%) and SCAR2 based on Rv1508c coding gene showed the secondary high positive rate (99.7%). SCAR3 (based on Rv2135c coding gene) showed relatively high deletion rate (3.2%) in the three SCAR markers, but less than the deletion rate of IS6110 (8%).

Moreover, the three SCAR markers were also evaluated in clinical sputum specimens. After resolution of the discrepant analysis by comparison of Real-time PCR in sputum specimens with bacteriological and clinical data, the sensitivities and specificities, positive predictive value (PPV) and negative predictive value (NPV) of all NAA tests were higher than those of bacteriological detection (acid-fast staining of bacilli smears and mycobacterial culture). In four NAA tests, IS6110 and SCAR3 showed the highest PPV (100%) and low NPV (70% and 68.8%, respectively), and SCAR1 and SCAR2 showed the relatively high PPV and NPV (97% and 82.6%, 95.6% and 88.8%, respectively).

The ideal target sequence should be present in all strains of the *M. tuberculosis* complex to avoid false-negative reactions, and absent in all other mycobacterium species to eliminate the chance of false-positive results. If not, the choice of targets with a high degree of sequence conservation or a multisite system based on more targets is necessarily required. The SCAR1 and SCAR2 markers with a high degree of sequence conservation may represent efficient and promising alternatives as NAA test targets in identification of MTB. SCAR3 and IS6110 showed relatively high deletion rate, but they had the high PPV. Maybe, they can be as alternative targets for the development of a multisite system to effectively detect MTB in samples.
